# Mortality among tuberculosis patients under DOTS programme: a historical cohort study

**DOI:** 10.1186/s12889-016-3557-0

**Published:** 2016-08-25

**Authors:** Yeshiwork Beyene, Berhanu Geresu, Assefa Mulu

**Affiliations:** 1 Department of Nursing, College of Medicine and Health Sciences Wollo University, Dessie, Ethiopia; 2 Department of Pharmacy, College of Medicine and Health Sciences, Wollo University, Dessie, Ethiopia

**Keywords:** Mortality, Tuberculosis, Survival, HIV, Dessie Referral Hospital

## Abstract

**Background:**

In high human immunodeficiency virus (HIV) prevalence population, tuberculosis (TB) is the leading cause of morbidity and mortality. HIV is driving the TB epidemic in many countries, especially those in sub-Saharan Africa. We assessed the survival time and predictors of mortality among tuberculosis patients under directly observed treatment, short course (DOTS) strategy in Dessie Referral Hospital tuberculosis clinic, Northeast Ethiopia.

**Method:**

A historical cohort design was utilized to assess survival time and determinants of mortality. A total of 1260 records of patients who started ant-tuberculosis treatment from January 2006 up to December 2010 were analyzed. Survival curves were estimated using Kaplan–Meier and were compared using the Log-rank test. The Cox proportional hazard model was used to assess the relationship between baseline variables and mortality.

**Results:**

Out of the 1260 registered patients, 117 (9.3 %) died over the entire follow-up period. Among those died, 113 (18 %) were HIV positive and 4 (0.6 %) were HIV negative. The 1260 patients contributed a cumulative total of 634.25 person‑years observation.

**Conclusion:**

The mortality of HIV positive tuberculosis patients was higher than those of HIV negative patients and the use of cotrimoxazole preventive therapy increased the survival time of patients.

## Background

Tuberculosis (TB) remains a major global health problem. It causes ill-health among millions of people each year and ranks as the second leading cause of death from an infectious disease worldwide, after HIV. In 2014, 6 million new cases of TB were reported to the World Health Organization (WHO) and TB killed 1.5 million people (1.1 million HIV-negative and 0.4 million HIV-positive). Despite the fact that nearly all cases can be cured, TB remains one of the world’s biggest threats [1]. The synergy between TB and HIV is strong; in high HIV prevalence population, TB is a leading cause of morbidity and mortality, and HIV is driving the TB epidemic in many countries, especially those in sub-Saharan Africa [2].

Ethiopia is one of the 22 high burden countries and TB remains one of the leading causes of mortality. According to the 2014 WHO report, the prevalence and incidence of all forms of TB are 211 and 224 per 100,000 of the population, respectively. Excluding HIV related deaths, in 2013 TB mortality was estimated to be 32 per 100,000 of the population. About 13 % of all new TB cases are also HIV co-infected. Moreover, Ethiopia is one of the high TB/HIV burden countries. Among TB patients with known HIV status, about 11 % were HIV co-infected [1].

In Ethiopia a standardized TB prevention and control programme, incorporating Directly Observed Treatment, Short Course (DOTS), was started in 1992. The DOTS strategy has been subsequently scaled up in the country and implemented at national level [3]. The scale of the global TB epidemic demands urgent and effective action. It is very important in TB control to detect the disease as early as possible and to ensure that those diagnosed complete their treatment and get cured [4].

In 1995, WHO set a target of 85 % treatment success among new sputum smear-positive cases [4]. Identification of factors for treatment failure is important in reducing TB spread, morbidity and mortality in affected individuals and may help in contributing to the achievement of the treatment targets.

Understanding the predictors of mortality for TB-HIV co-infected patients in the local context is critical for Ethiopia to improve TB-HIV co-infected patients’ co-management. The survival time of TB patients can be affected by different factors. Studies showed that antiretroviral therapy (ART) status, cotrimoxazole preventive therapy (CPT) status and type of TB diagnosis were independent predictors of mortality. Initiation of ART and CPT as well as extra pulmonary TB type decreased risk of mortality in TB/HIV co-infected individuals [5]. In resource-poor settings limited data exist both on treatment results and on how to carry out such interventions. As a result, the existing treatment guidelines and recommendations are based on data from the developed world. Studying the survival patterns will help in identifying the risk factors for mortality in these patients and planning effective interventions to further reduce death rates. Therefore, the aim of this study was to assess the survival time and predictors of mortality among TB patients under DOTS strategy in Dessie Referral Hospital (DRH) TB clinic, Northeast Ethiopia.

## Methods

The study was conducted in DRH TB clinic, Northeast Ethiopia from January 1–30, 2014. DRH has a catchment population of seven million and has a total bed size of 200 with 185 health professionals (13 specialists, 14 general practitioners, 3 health officers, 6 anesthetists, 99 nurses, 12 midwifes, 12 pharmacy professionals, 12 medical laboratory professionals, 5 X-ray technicians and 2 environmental health professionals. The TB clinic is one of the hospital segments serving 300 patients on average per annum.

A historical cohort design was utilized to assess survival time and predictors mortality. Based on HIV status, TB patients was categorized into “HIV positive” and “HIV negative” cohorts and were retrospectively followed until the time of the outcome (death) or censoring. Survival time was measured from the date of initiation of therapy to death or to the last follow-up. Individuals who died were considered as failures, those who remained alive until the end of the treatment or dropped out were considered as censored. A total of 1260 out of 1375 patient record cards of TB patients who started ant-TB treatment from January 2006 up to December 2010 in DRH TB clinic were included and retrospectively followed for an additional one year.

The independent variables were age, sex, residence, baseline weight, HIV status, type of TB, and CPT. The outcome variable was survival time.

Structured questionnaire developed using standardized TB entry and follow up form employed by the TB clinic was utilized to extract the required data from patient records. The data was collected by reviewing registers, follow up form and patients’ card. Two nurses working at the TB clinic of DRH were recruited and trained about methods of data collection for two days. Data quality was controlled by designing the proper data collection materials and through continuous supervision. All completed data collection forms were examined for completeness and consistency during data management and analysis. The data was entered and cleaned by a data clerk and the principal investigator before analysis.

The survival time was calculated using the time interval between the date of anti-TB initiation and the date of event (death) or censoring. The Kaplan-Meier model was used to estimate the survival probability after anti-TB initiation, and the Log-rank test was used to compare survival curves. The Cox proportional hazard model was used to assess the relationship between baseline variables and mortality. Descriptive statistics and Cox regression were conducted using SPSS version 16.

Ethical clearance was obtained from College of Medicine and Health Sciences, Wollo University Institutional Review Committee (IRC), and permission was sought from DRH.

## Results

### Demographic and clinical characteristics

In this retrospective document analysis from January 2006 up to December 2010, socio-demographic and medical information of 1260 registered TB patients was summarized. Out of the total study participants 781(62 %) were males and 1206 (95.7 %) were from urban areas. Half (50.1 %) of the study subjects were HIV negative, 16.4 % were smear positive pulmonary TB, 39.8 % were smear negative pulmonary TB and 43.7 % were extra pulmonary TB patients. Majority of the study participants (83.6 %) were new cases (Table 1).

**Table 1:** Baseline characteristics of TB patients treated at DRH from January 2006 – December 2010

Variables	HIV status		Total
	Positive (*n =* 629)	Negative (*n =* 631)	
Gender			
Male	341 (54.2)	440 (69.7)	781 (62.0)
Female	288 (45.8)	191 (30.7)	479 (38.0)
Age group			
< 20	83 (13.2)	80 (12.7)	163 (12.9)
20–29	110 (17.5)	288 (45.6)	398 (31.6)
30–39	274 (43.6)	148 (23.5)	422 (33.5)
40–49	101 (16.1)	36 (5.7)	137 (10.9)
> 49	61 (9.7)	79 (12.5)	140 (11.1)
Residence			
Urban	600 (95.4)	606 (96.0)	1206 (95.7)
Rural	29 (4.6)	25 (4.0)	54 (4.3)
Baseline weight			
< 20	43 (6.8)	31 (4.9)	74 (5.9)
20–29	16 (2.5)	3 (0.5)	19 (1.5)
30–39	45 (7.2)	63 (10.0)	108 (8.6)
40–49	195 (31.0)	136 (21.6)	331 (26.3)
> 49	330 (52.5)	398 (63.1)	728 (57.8)
CPT initiated			
No	186 (29.6)	581 (92.1)	767 (60.9)
Yes	443 (70.4)	50 (7.9)	493 (39.1)
ART initiated			
No	348 (55.3)	631 (100)	979 (77.7)
Yes	281 (44.7)	0 (0.0)	281 (22.3)
Type of TB			
Smear positive	67 (10.7)	140 (22.2)	207 (16.4)
Smear negative	291 (46.3)	211 (33.4)	502 (39.8)
EPTB	271 (43.1)	280 (44.4)	551 (43.7)
Treatment category			
New	539 (85.7)	514 (81.5)	1053 (83.6)
Retreatment	90 (14.3)	117 (18.5)	207 (16.4)
Patient status			
Censored	516 (82.0)	627 (99.4)	1143 (90.7)
Died	113 (18.0)	4 (0.6)	117 (9.3)

*CPT* Cotrimoxazole preventive therapy, *ART* Antiretroviral therapy, *TB* Tuberculosis, *EPTB*-Extrapulmonary TB

### Survival status of the study subjects

**Fig. 1:**
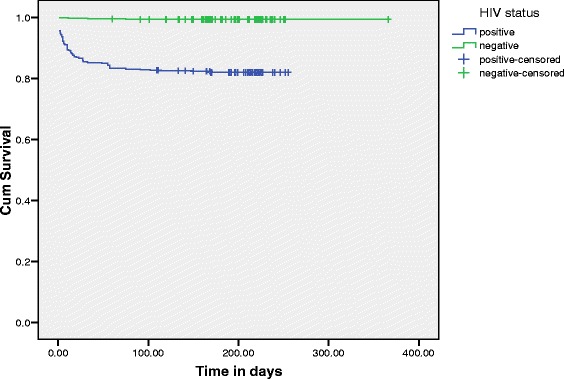
Survival curve by HIV status of TB patients (*n =* 1260) treated at DRH from January 2006 – December 2010. Statistical analysis was done using the Kaplan-Meier model to estimate the survival probability after anti-TB initiation, and the Log rank test was used to assess the relationship between HIV status and mortality. The result showed that the survival time in days of HIV negative cohorts was significantly (Log rank test = 112.423, *p <* 0.001) higher than those of HIV positive cohorts. Statistical significance was set at *p <* 0.05

Of 629 HIV positive and 631 HIV negative TB patients, 113 (18 %) of the HIV positive and 4 (0.6 %) of the HIV negative died and were treated as failure in the analysis. The remaining 516 (82.0 %) HIV positive and 627 (99.4 %) HIV negative patients became censored during the follow up period (Table 1). The 1260 patients contributed a cumulative total 634.25 person‑years observation, which gave rise to the overall mortality rate of 18.4 per 100 person-years of follow up per annum (117 died over the entire person-years of follow up). The median and the mean survival times were 210 and 183.7 days, respectively. The log-rank test was conducted to check for existence of any significant differences in survival experience among various levels of the categorical variables included in the study. The result in Fig. 1 manifest a significant (Log rank test = 112.423, *p* < 0.001) differences among HIV positive and HIV negative cohorts.

### Predictors of mortality

The relationship between the main baseline variables and the risk of death was analyzed using a Cox proportional hazard regression model. The result showed that gender, age, residence, weight, CPT, type of TB and HIV status were all significantly associated with death of TB patients during the period of TB treatment (Table 2). Compared to HIV positive patients, the hazard ratio (HR) of dying from TB decreased significantly by 99 % in HIV negative TB patients; adjusted hazard ration (AHR) = 0.01, 95 % CI: [0.003, 0.027]. Mortality risk among female TB patients increases significantly by about two fold compared to males; AHR = 2.360, 95 % CI: [1.518, 3.671]. According to the result the risk of dying significantly reduced in patients receiving CPT by 76.6 % compared to those not receiving CPT; AHR = 0.234, 95 % CI: [0.145, 0.377].

**Table 2:** Predictors of mortality among TB patients treated at DRH from January 2006 – December 2010

Variables	Status		Adjusted hazard ratio (95 % CI)	*p-*value
	Censored	Died		
Gender				
Male	727	54	1	
Female	416	63	2.360 (1.518, 3.671)^a^	0.000
Age group				
< 20	129	34	1	
20–29	366	32	1.317 (0.629, 2.755)	0.465
30–39	401	21	0.335 (0.156, 0.722)^a^	0.005
40–49	120	17	1.029 (0.442, 2.394)	0.948
> 49	127	13	1.687 (0.649, 4.385)	0.283
Residence				
Urban	1114	92	1	
Rural	29	25	4.230 (2.508, 7.136)^a^	0.000
Baseline weight				
< 20	54	20	1	
20–29	19	0	0.000 (0.000, 0.000)	0.957
30–39	99	9	1.103 (0.420, 2.894)	0.843
40–49	291	40	0.811 (0.332, 1.980)	0.646
> 49	680	48	0.389 (0.178, 0.851)^a^	0.018
HIV status				
Positive	516	113	1	
Negative	627	4	0.010 (0.003, 0.027)^a^	0.000
CPT initiated				
No	688	79	1	
Yes	455	38	0.234 (0.145, 0.377)^a^	0.000
ART initiated				
No	891	88	1	
Yes	252	29	0.694 (0.414, 1.163)	0.166
Type of TB				
Smear positive	192	15	1	
Smear negative	472	30	0.417 (0.207, 0.837)^a^	0.014
EPTB	479	72	1.149 (0.595, 2.222)	0.679
Treatment category				
New	944	109	1	
Retreatment	199	8	0.438 (0.191, 1.005)	0.051

*CPT* Cotrimoxazole preventive therapy, *ART*-Antiretroviral therapy, *TB* Tuberculosis, *EPTB* Extrapulmonary TB

^a^Significant at α = 0.05

## Discussion

WHO defines TB mortality as the number of TB cases dying during treatment, regardless of the cause. Death caused by TB is preventable and management can be modified at the time of highest risk for death if known [6]. It is now recognized that both TB and HIV contribute to each other’s progress. Patients co-infected with TB and HIV have higher mortality rates in comparison with those infected with one or the other. In several case series, the main predictor for survival was the prompt initiation of effective anti-tuberculosis treatment, ART, CD_4_ count, site of the disease, and other previous or concurrent opportunistic infections caused by immunosuppression [7–9].

Of the 1260 registered patients, 117 (9.3 %) died over the entire follow-up period. The mortality in our study was higher than the report in Addis Ababa, Ethiopia [10] and the nationwide mortality in Ethiopia [3], but lower than those reported in previous studies including 10.0 % in Gondar, Ethiopia [11], and 14 % in Vaud County, Switzerland [12]. Similarly, the study has demonstrated significant difference on the prevalence of death between HIV positive and HIV negative TB patients. Of 629 HIV positive and 631 HIV negative TB patients, 113 (18 %) of the HIV positive and 4 (0.6 %) of the HIV negative died. Similar to the current study, previous studies [13] reported higher overall death rates among HIV positive TB patients than HIV negative TB patients.

HIV infection is the primary reason for the failure to meet tuberculosis control targets (at least 85 % cure rate among new sputum smear positive TB cases) in countries with high HIV infection. This is attributable to factors such as over diagnosis of sputum smear negative TB, under diagnosis of sputum smear-positive TB, low cure rates, high morbidity, mortality and default rates during treatment, and atypical clinical presentation of TB in HIV infected patients. Consequently, HIV infection leads to diagnostic challenges and delays in identifying TB that profoundly impacts treatment outcome [14].

The relationship between the main baseline variables and the risk of death was analyzed using a Cox proportional hazard regression model. In our study not initiating CPT was associated with high risk of mortality. In line with this, studies from Northwest Ethiopia [5] South India [15] and Sub-Saharan Africa [16] showed that not taking CPT was significantly associated with mortality. CPT is a simple well tolerated and cost effective intervention which can extend and improve the quality of life for people living with HIV/AIDS including those on ART. CPT is associated with a 25–46 % reduction in mortality among individuals infected with HIV in sub-Saharan Africa even in areas of high bacterial resistance to the antibiotic [17, 18].

Previous studies found that TB-HIV co-infected patients who took ART during TB treatment had a lower risk of death. They also demonstrate the positive impact of ART on the survival outcomes among TB-HIV co-infected patients, including successful immune restoration and reductions in morbidity and mortality [5, 19]. In our study we did not find any significant difference in mortality between the on ART and non-ART cohorts, which is in agreement with previous studies [13]. This might be associated with delayed initiation of ART. The optimal time to initiate ART in patients with HIV-associated tuberculosis has been the subject of intense debate. Concerns about early initiation of ART include a high pill burden, pharmacological interactions, overlapping toxicities and the immune reconstitution inflammatory syndrome (IRIS) [20]. Conversely, delayed initiation of ART may be associated with HIV disease progression and death [21]. Previous retrospective studies [22, 23] showed that delaying ART initiation until after completion of TB therapy was associated with increased mortality.

Previous TB treatment and multiple drug‑resistant TB (MDR‑TB) are considered significant risk factors for decease. It is known that patients previously treated for TB have a higher risk of presenting MDR TB and of dying than new TB patients [24]. There are also reports supporting the idea that failure or relapses were not associated with an increased risk of death [25]. This study also adds to the literature that treatment category (new or retreatment) was not significantly associated with mortality in the adjusted model, this might be due to late presentation of patients to the hospital and advanced disease progress.

Our study was done retrospectively to find out the common risk factors associated with mortality that was considered one of the limitations of the survey. The data have been extracted from medical records of patients who have been already visited and registered at the hospital, so it may be subjected to selection bias. The other limitation of the survey was the fact that in many TB patients, multiple causes of death may act simultaneously, so the specific cause of death may not be determined accurately.

## Conclusions

In summary, results from this study indicate that the mortality of HIV positive TB patients was higher than those of HIV negative TB patients and use of CPT can prolong the survival time of TB patients. The decrease in mortality among TB/HIV patients requires public health interventions and the enhancement of existing control programs to improve both prevention and treatment. Interventions should be directed at modifiable risk such as treatment of opportunistic infections.

## References

[1] World Health Organization (2015). Global Tuberculosis control report.

[2] Federal Minister of Health (2007). Implementation Guideline for TB/HIV Collaborative Activities in Ethiopia.

[3] Federal Ministry of Health (2012). Guidelines for clinical and programmatic management of TB, TB/HIV and leprosy in Ethiopia.

[4] World Health Organization (2010). Treatment of tuberculosis: Guidelines for National Programmes.

[5] Sileshi B, Deyessa N, Girma B, Melese M, Suarez P (2013). Predictors of mortality among TB-HIV Co-infected patients being treated for tuberculosis in Northwest Ethiopia: a retrospective cohort study. BMC Infect Dis.

[6] World Health Organization (1994). Framework for effective tuberculosis control.

[7] Ackah AN, Coulibaly D, Digbeu H, Diallo K, Vetter KM, Coulibaly IM, Greenberg AE, De Cock KM (1995). Response to treatment, mortality, and CD4 lymphocyte counts in HIV-infected persons with tuberculosis in Abidjan, Cote d’Ivoire. Lancet.

[8] Whalen C, Horsburgh CR, Hom D, Lahart C, Simberkoff M, Ellner J (1995). Accelerated clinical course of HIV infection after tuberculosis. Am J Respir Crit Care Med.

[9] Shafer RW, Bloch AB, Larkin C, Vasudavan V, Seligman S, Dehovitz JD, DiFerdinando G, Stoneburner R, Cauthen G (1996). Predictors of survival in HIV-infected tuberculosis patients. AIDS.

[10] Getahun B, Ameni G, Biadgilign S, Medhin G (2011). Mortality and associated risk factors in a cohort of tuberculosis patients treated under DOTS programme in Addis Ababa, Ethiopia. BMC Infect Dis.

[11] Tessema B, Muche A, Bekele A, Reissig D, Emmrich F, Sack U (2009). Treatment outcome of tuberculosis patients at Gondar University Teaching Hospital, Northwest Ethiopia. A five - year retrospective study. BMC Publ Health.

[12] Zellweger JP, Coulon P (1998). Outcome of patients treated for tuberculosis in Vaud County, Switzerland. Int J Tuberc Lung Dis.

[13] Shaweno D, Worku A (2012). Tuberculosis treatment survival of HIV positive TB patients on directly observed treatment short-course in Southern Ethiopia: A retrospective cohort study. BMC Res Notes.

[14] Mahajan V, Verma SK. HIV-Tuberculosis co-infection. The Internet Journal of Pulmonary Medicine 2008, http://www.ispub.com Accessed Jul 12 2013.

[15] Vijay S, Kumar P, Chauhan LS, Narayan Rao SV, Vaidyanathan P (2011). Treatment outcome and mortality at one and half year follow-Up of HIV infected TB patients under TB control programme in a District of South India. PLoS One.

[16] Harries AD, Zachariah R, Lawn SD (2009). Providing HIV care for co-infected tuberculosis patients: a perspective from sub-Saharan Africa. Int J Tuberc Lung Dis.

[17] Badri M, Ehrlich R, Wood R, Maartens G (2001). Initiating co-trimoxazole prophylaxis in HIV-infected patients in Africa: an evaluation of the provisional WHO/UNAIDS recommendations. AIDS.

[18] World Health Organization (2009). Guideline on cotrimoxazole prophylaxis for HIV related infections among children, adolescent & adults.

[19] Cain KP, Anekthananon T, Burapat C, Akksilp S, Mankhatitham W, Srinak C, Nateniyom S, Sattayawuthipong W, Tasaneeyapan T, Varma JK (2009). Causes of death in HIV-infected persons who have tuberculosis, Thailand. Emerg Infect Dis.

[20] Stockdale AJ, Nkuranga J, Török ME, Faragher B, Lalloo DG (2013). Initiation of Antiretroviral Therapy in HIV-Infected Tuberculosis Patients in Rural Kenya: An Observational Study. Trop Med Int Health.

[21] Lawn SD, Churchyard G (2009). Epidemiology of HIV-associated tuberculosis. Curr Opin HIV AIDS.

[22] Manosuthi W, Chottanapand S, Thongyen S, Chaovavanich A, Sungkanuparph S (1999). Survival rate and risk factors of mortality among HIV/tuberculosis coinfected patients with and without antiretroviral therapy. J Acquir Immune Defic Syndr.

[23] Velasco M, Castilla V, Sanz J, Gaspar G, Condes E, Barros C, Cervero M, Torres R, Guijarro C (1999). Effect of simultaneous use of highly active antiretroviral therapy on survival of HIV patients with tuberculosis. J Acquir Immune Defic Syndr.

[24] Tocque K, Convrey RP, Bellis MA, Beeching NJ, Davies PD (2005). Elevated mortality following diagnosis with a treatable disease: tuberculosis. Int J Tuberc Lung Dis.

[25] Lefebvre N, Falzon D (2008). Risk factors for death among tuberculosis cases: analysis of European surveillance data. Eur Respir J.

